# Dividing the Internal Rectus Muscle for the Cure of Squinting

**Published:** 1840-07

**Authors:** 


*Dividing the Internal Rectus Muscle for the cure of Squinting.*—William James Egan, aged 10, was born with his eyes perfectly straight. When he was two years of age he suffered from convulsions, and after a more severe one than usual the strabismus took place.

*Present state.*—His left eye turned deeply inwards, with a slight degree of obliquity upwards; its sight is not so powerful as that of the right; there is a slight degree of opacity of the inner circumference of the cornea, and the organ is more sunken than its fellow. With much exertion the patient can evert the eye to the extent of about four lines.

With the assistance of Mr. Downing, Mr. Bailey, Mr. Earles, and Mr. Snow, I performed this operation as follows: The eyelids were held apart simply by means of the fingers; I seized the inner conjunctiva with a small sharp hook, and divided this membrane from below upwards, with a fine narrow bladed knife. At the instant of doing this the eye forcibly turned more inwards, which retarted the operation a a few moments: keeping the hook still fixed in the inner segment of the divided conjunctiva, I allowed the lids to cover the globe, and a few drops of blood were wiped away by means of a sponge and cold water. Exposing the eye again by simply elevating the upper, and depressing the inner lids with the fingers of two assistants, I readily exposed the incision of the conjunctiva, and having separated the connecting reticular tissue by a blunt probe, as in the other cases; I introduced the blunt hook, and with much facility passed it from below upwards, beneath the inner rectus muscle, and drawing it forwards, I divided its tendon with a curved scissors.

The tendon of the muscle was unusually thick and strong, far different from the appearance which it presented in the other cases; it *grated* beneath the blades of the scissors upon dividing it, which, being accomplished, the eye became instantaneously straight.

The whole operation only occupied two minutes.

In this case the speculum was dispensed with, and the only instruments used were a hook, a knife, a probe, and a scissors.

After the operation a cold bread and water poultice was applied to the eye, a powder, consisting of Jame's powder and calomel, was given, and the boy was put to bed.

This little patient evinced great strength. I explained to him beforehand the object of the operation, and saw he could assist us by everting his eye as much as possible, and which he did at the time it was most needed.

*Remarks.*—Were the operation of dividing the muscles of the human eye for the cure of strabismus, attended with danger to the organ of vision, with consequences of even a much less serious nature, the propriety of its performance might justly be regarded as questionable. But when it is considered that no bad consequences have followed this interesting operation—that the patient suffers but little during its performance—and that, in the cases to which it is applicable, the most gratifying success has attended it; its extensive application to the removal of strabismus cannot be too forcibly insisted upon.—*London Lancet. American Med. Int. June* 15, 1840.

